# Supplementary figures and images for: A computational method to aid the design and analysis of single cell RNA-seq experiments for cell type identification
Source: BMC Bioinformatics. 2019 Jun 6;20(Suppl 11):275. doi: 10.1186/s12859-019-2817-2 (PMC6551246; doi:10.1186/s12859-019-2817-2)

**fC4, 1000 cells**

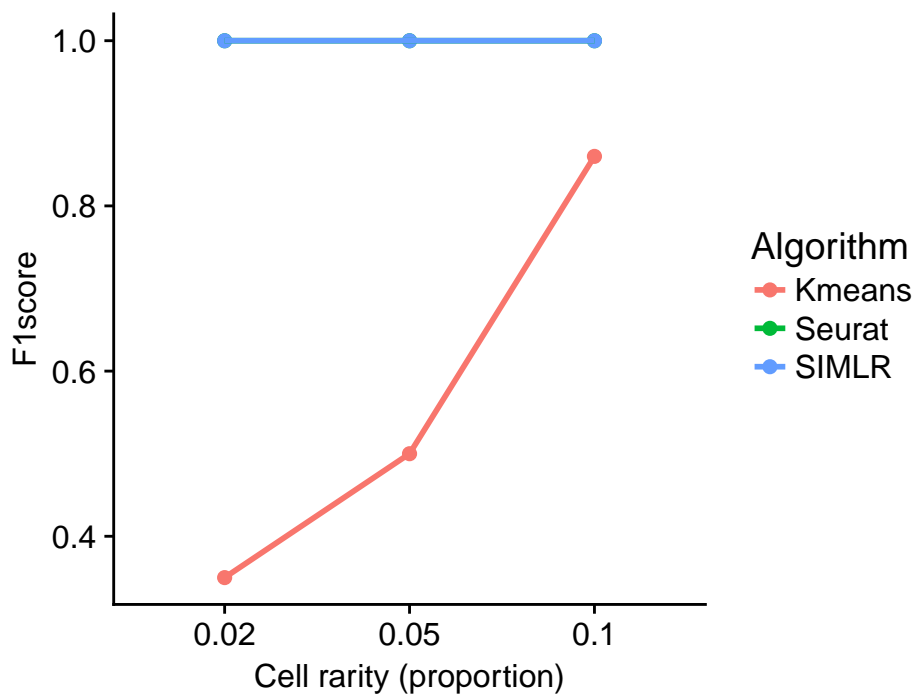

**fC4, 2000 cells**

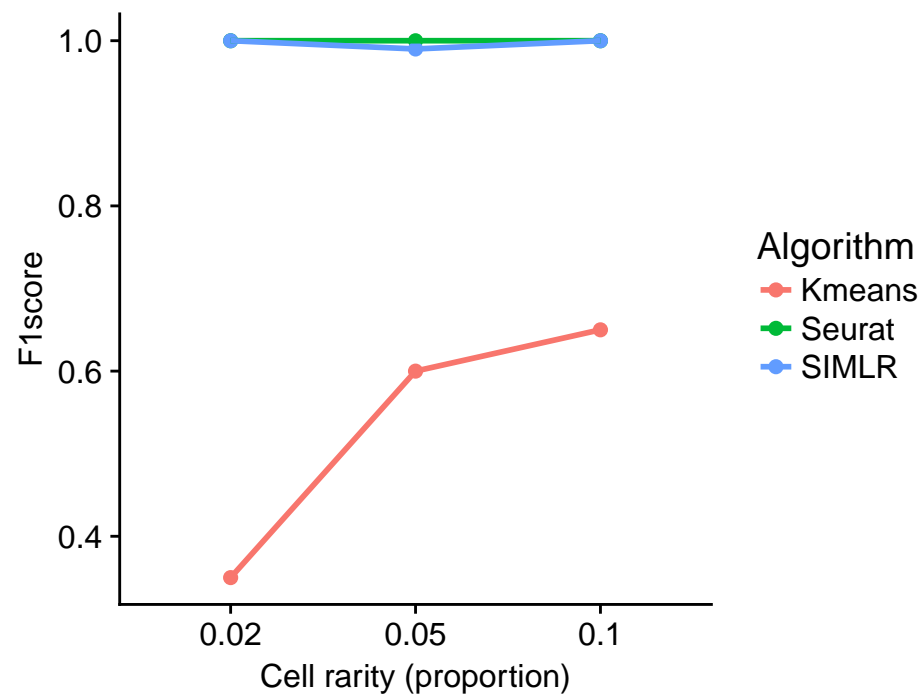

**fC4, 3000 cells**

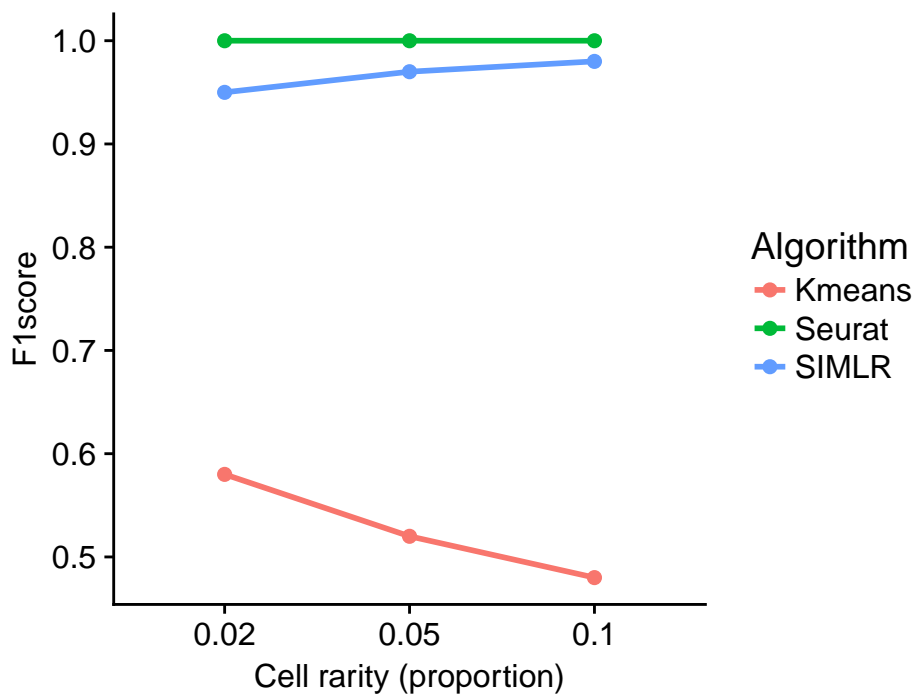

Supplement: Supplementary file 1 — Performance of different single cell algorithms at different cell proportions. F1score was calculated at cell rarity proportions of 0.02, 0.05 and 0.1 containing 4 foldchange upregulated marker genes for 1000, 2000 and 3000 single cells datasets. X-axis represents the cell rarity proportions while y-axis represents F1score. (PDF 5 kb) [file 12859_2019_2817_MOESM1_ESM.pdf]

**fC8, 1000 cells**

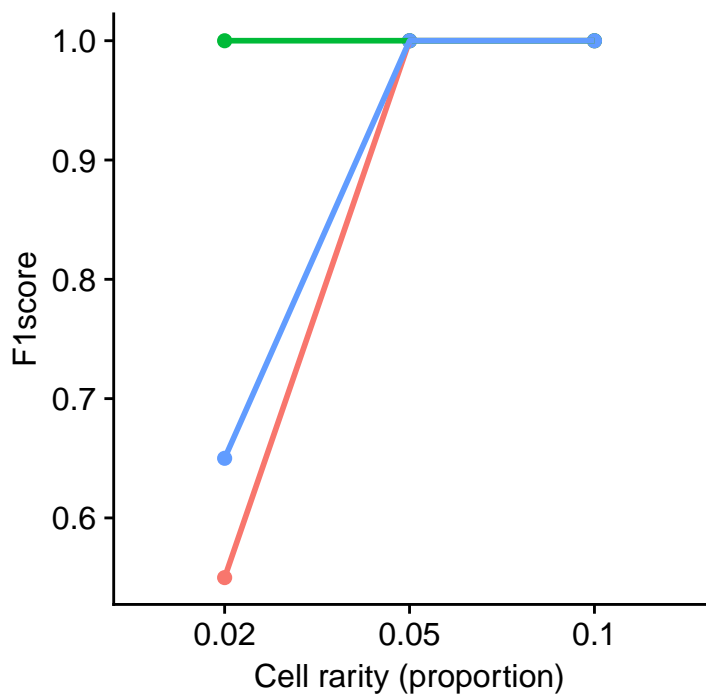

**fC8, 2000 cells**

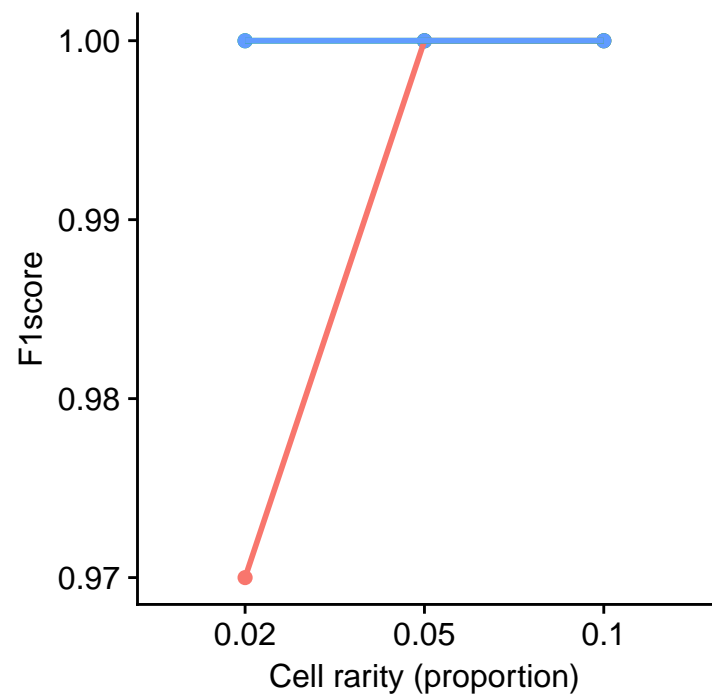

**fC8, 3000 cells**

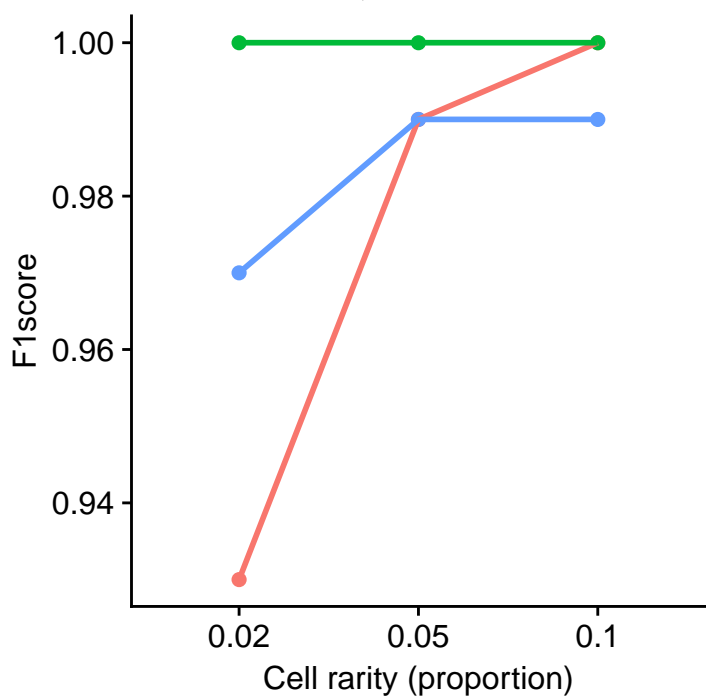

Supplement: Supplementary file 2 — Performance of different single cell algorithms at different cell proportions. F1score was calculated at cell rarity proportions of 0.02, 0.05 and 0.1 containing 8 foldchange upregulated marker genes for 1000, 2000 and 3000 single cells datasets. X-axis represents the cell rarity proportions while y-axis represents F1score. (PDF 5 kb) [file 12859_2019_2817_MOESM2_ESM.pdf]
